# Prevalence and associated factors of hemorrhoids among adult patients visiting the surgical outpatient department in the University of Gondar Comprehensive Specialized Hospital, Northwest Ethiopia

**DOI:** 10.1371/journal.pone.0249736

**Published:** 2021-04-20

**Authors:** Anteneh Ayelign Kibret, Mohammed Oumer, Abebe Muche Moges

**Affiliations:** Department of Human Anatomy, School of Medicine, College of Medicine and Health Sciences, University of Gondar, Gondar, Ethiopia; German Centre for Neurodegenerative Diseases Site Munich: Deutsches Zentrum fur Neurodegenerative Erkrankungen Standort Munchen, GERMANY

## Abstract

**Introduction:**

Hemorrhoidal disease is a very common benign anorectal disease. It affects millions of people around the world, and represent a major medical and socioeconomic problem. However, studies that determine the magnitude and risk factors are limited. Therefore, the aim this study is to assess the prevalence and associated factors of hemorrhoid among adult patients visiting the surgical outpatient department at the University of Gondar Comprehensive Specialized Hospital (UoGCSH) Northwest Ethiopia.

**Methods:**

An institution-based cross-sectional study was conducted from February to May 2020. A systematic random sampling technique was used to select a total of 403 participants. The data were collected then entered using EPI DATA version 3.1 and exported to the STATA 14 for analysis. Bivariable and multivariable logistic regression analysis were performed. Adjusted odds ratio (AOR) with 95% confidence interval was used as a measure of association. Variables having P-value < 0.05 from the multivariable analysis were considered to have a significant association with the outcome.

**Result:**

Out of the 403 study participants, 13.1% (95%CI; 10.1, 16.8) had hemorrhoids. Constipation (AOR = 4.32, 95% CI; 2.20, 8.48) and BMI ≥25kg/m^2^ (AOR = 2.6, 95% CI; 1.08, 6.23) had a statistically significant association with hemorrhoid.

**Conclusion:**

The overall prevalence of hemorrhoid was high and its prevalence was higher in male subjects. Constipation and being overweight were found to increase the odds of having hemorrhoids. Screening for early identification and intervention of hemorrhoids, especially for risk groups is better to be practiced by health professionals.

## Introduction

Hemorrhoid is the anastomoses between the superior rectal artery and the superior, middle, and inferior rectal vein that surround the distal rectum and anal canal. It is a distal displacement and venous distention of the hemorrhoidal cushions [1, 2]. Based on location, hemorrhoids are usually classified as internal and external hemorrhoids. Internal hemorrhoids arise above the dentate line and are covered by columnar epithelium, while external hemorrhoids arise below the dentate line and are covered by squamous epithelium [3–5]. Patients with hemorrhoids are usually asymptomatic but the common symptoms are bleeding with or without defecation, a swelling, mild discomfort or irritation and pruritus ani [6, 7]. Though, some patients need to undergo surgery, many hemorrhoid patients can successfully be treated with conservative medication and ointments [8]. The pathogenesis of hemorrhoids is a weakening of the anal cushion leads to descent or prolapse of the hemorrhoids and spasm of the internal sphincter [9, 10]. In the United States, hemorrhoid disease is the fourth leading outpatient gastrointestinal diagnosis, accounting for 3.3 million ambulatory care visits [5]. The estimated worldwide prevalence of hemorrhoids in the general population is to be 4.4% [11]. Globally, various studies were conducted to assess the prevalence and associated factors of hemorrhoids. The prevalence of hemorrhoid is higher in Australia (38.93%) which is followed by Israel (16%) and Korea (14.4%) [12–14].Very few attempts have been made to assess the prevalence of hemorrhoids in Africa. The prevalence of hemorrhoid among Egypt patients subjected to colonoscopy was 18% [15].

Studies conducted elsewhere indicated that inadequate dietary fiber, constipation, diarrhea, hypertension, high body mass index (BMI), pregnancy and old age are the commonly identified risk factors for the development of hemorrhoids [1, 13, 14].

Hemorrhoids are now considered a major cause of morbidity and impose both economical and social impact on society [12]. It has social impact, as it is inter-linked to lifestyles, such as interpersonal, and impacted by food and hygienic and sexual habits, and also has economic burden on health systems in direct costs and working days lost [16]. The hemorrhoidal disease creates physical and psychological discomfort and significantly affects the quality of life of the patients due to its sensitive symptoms such as anal bleeding, pain and itching sensation [12, 17]. Besides, hemorrhoids hinder patient’s ability to live normally and work efficiently even after management due to its frequent recurrence, incomplete elimination of discomfort and postoperative pain [14]. The most common and serious complications of hemorrhoids include perianal thrombosis and incarcerated prolapsed internal haemorrhoids with subsequent thrombosis [18].

Reports on the magnitude and risk factors of hemorrhoids have paramount importance to the policymakers, clinical practitioners, and the society at large. In spite of sever clinical and social impacts, there is no documented evidence in Ethiopia so far. The present study is aimed to determine the prevalence and associated factors of hemorrhoids among patients visiting the surgical outpatient department (OPD) at the UoGCSH, Northwest Ethiopia.

## Methods

### Study design and setting

An institution based cross-sectional study design was conducted from February to May 2020 G.C among adult surgical patients who visited the surgical outpatient department (OPD) at the UoGCSH. The hospital was found in1954 and it is located in the Central Gondar administrative zone, Amhara National Regional State, which is about 750 km Northwest of Addis Ababa (the capital city of Ethiopia). According to the 2015 population projection of major cities in Ethiopia, the total population size of Gondar town was estimated to be 323,900. Currently, Gondar town has one Referral Hospital and eight government Health Centers. University of Gondar Comprehensive Specialized Hospital is a teaching hospital, which serves more than five million people of the North Gondar zone and peoples of the neighboring zones. It is estimated that around 21,000 patients visit the surgical OPD per year.

### Population and sample size determination

The source population of the study was all adult patients above 18 years’ old who visited the surgical OPD at the UOG Comprehensive Specialized Hospital. The study population was all adult patients above 18 years’ old who visited the surgical OPD during the time of data collection in the UOG Comprehensive Specialized Hospital. Patients who were unable to communicate, mentally ill, and severely ill were excluded from the study. The sample size was determined using a single population proportion formula, by using a 95% confidence interval, 0.05 margin of error, 5% non-response rate. As far as our search is concerned, there was no previous study conducted in the area and the expected proportion of hemorrhoids was considered to be 50%. Therefore, the final sample size was 403 and participants were selected using a systematic random sampling technique with skipping intervals of three.

### Variables and data collection procedures

The outcome variable of this study was hemorrhoids. Patients were diagnosed based on history and anorectal examination which includes inspection, digital examination and anoscopy. Grading of hemorrhoids was documented and classified according to the international classification recommended by Banov et al: Hemorrhoids that do not prolapse and appear as a bulge into the lumen of the anal canal with or without bleeding (grade I). Hemorrhoids that prolapse and reduce spontaneously (grade II). Hemorrhoids that require digital reduction of prolapsed tissue (grade III).Hemorrhoidal piles prolapse is irreducible(grade IV) [12]. The first group of factors assessed was socio-demographic characteristics including age, sex, residence, educational status, occupation, marital status, and average monthly income. The second was clinical factors which includes a Family history of hemorrhoids, constipation, body mass index (BMI), hypertension, fiber diet intake, and chronic diarrhea. The third group of characteristics assessed was Behavioral and obstetric factors mainly focused on Smoking, alcohol intake and parity. Blood pressure(BP) was measured three times in a sitting position using a standard mercury sphygmomanometer BP cuff with the appropriate cuff size that covers two-thirds of the upper arm after the participant rested for at least five minutes and no smoking or caffeine 30 minutes before measurement. The second and the third measurements were taken five-to-ten minutes after the first and the second measurement, respectively. Finally, the average of the three BP measurements was calculated to determine the BP status of the participant. An individual was diagnosed as hypertensive if systolic blood pressure (SBP) is ≥130mmHg or diastolic blood pressure(DBP) is ≥80 mmHg or previous diagnosis of hypertension or current use of the anti-hypertensive drug [19]. Constipation means unsatisfactory defecation characterized by infrequent stool, difficulty in defecation or both at least for previous 3 months [20]. Fiber diet intake was measured, if participants took fiber diet once in a week considered as they had a history of adequate fiber diet intake. Weight and height to calculate BMI and was taken using calibrated equipment and BMI was calculated by dividing weight in kg by height in meters square. BMI <18.5 kg/m^2^ was considered as underweight 18.5–24.9 kg/m^2^ as normal, 25–29.9 kg/m^2^ overweight and ≥30 kg/m^2^as obese [21].

The interviewer-administered questionnaire was adopted from different works of literature. The questionnaire was first developed in English and then translated into Amharic language and back to English and consistency was checked. The data was completed by trained five B. Sc nurses who were working at the surgical OPD using the Amharic version questionnaire. Data quality was controlled through the provision of one-day training to the data collectors. To evaluate the general approachability and feasibility of the questionnaire, a pretest was carried out using 10% of a sample size at Debretabor Primary Hospital. Hence, correction and modification were made to the questionnaire accordingly.

### Data processing and analysis

After data collection, each questionnaire was checked visually for completeness. Data were coded and entered using EPI DATA 3.1 version and exported to the STATA 14 for analysis. Data cleaning was done by identifying and correcting missed values and inconsistencies. Descriptive statistics like frequency, percentage, median, and Interquartile range (IQR) was done to describe the study population in relation to different variables. A Chi-square test was done for all variables to check the assumptions. The binary logistic regression model was fitted as a primary method of analysis. Variables having p-value ≤ 0.2 from the bivariable analysis were chosen as a candidate for the final multivariable logistic regression model and variables having p-value <0.05 were considered to have a significant association with the outcome variable. An adjusted odds ratio with 95% CI was used as a measure of association. The model goodness of fit was assessed by the Hosmer-Lemeshow test.

### Ethical issues

The study protocol was approved by the ethical review committee of the College of Medicine and Health Sciences, University of Gondar (Reference SOM 950/12 dated February 15, 2020). Verbal informed consent was taken from all respondents enrolled in the study. Since most of our participants were an able to read and write so that we were not able to take written consent. The verbal consent was taken after introducing the topic of the research to the study participants, initially the data collectors give a brief description about the main aim of the research and telling them there honest and genuine participation by responding to the questions prepared is very important and highly appreciated to attain this purpose. Finally enable the study participants to know about their response to personal question are confidential and also can have a right to not answer any question if they do not want to and stop the interview at any time. The Institutional Review Board (IRB) approved use of consent. To keep confidentiality, respondent’s names and other personal identifiers were not included. The collected data were password protected and properly discarded.

## Results

### Socio-demographic characteristics

In the present study, a total of 403 study participants were involved with a response rate of 100%. The median age of the participants was 38 years old (IQR: 28, 52). Both sexes had nearly equal frequency, 207 (51.3%) female subjects and more than half of the study participants 210 (52.15%) had no formal education. Of the participants, 135 (33.5%) were farmers and 290 (72%) were married. Almost half of the study participants 200 (49.6%) had an average monthly income less than 1210 ETB (Table 1).

**Table 1:** Socio-demographic characteristics of adult patients visiting surgical OPD at the UOG Comprehensive Hospital, Ethiopia, 2020 (n = 403).

Variable	Frequency	Percentage
**Sex**		
Male	196	48.7
Female	207	51.3
**Age**		
19–33	161	40.0
34–48	120	30.0
49–63	81	20.1
64–78	35	8.5
79–84	6	1.5
**Residence**		
Urban	220	54.6
Rural	183	45.4
**Occupation**		
Farmer	135	33.5
Merchant	31	7.7
Civil servant	58	14.4
Housewife	98	24.3
Student	38	9.4
Daily laborer	18	4.6
Others*	25	6.2
**Religion**		
Orthodox	388	96.2
Muslim	11	2.8
Protestant	4	1.0
**Educational status**		
No formal education	210	52.1
Primary education	42	10.4
Secondary education	63	15.7
College or above	88	21.8
**Marital status**		
Married	290	72
Divorced	28	7
Widowed	9	2.2
Single	76	18.6
**Average monthly income in ETB**		
<1210	200	49.6
1211–8970	194	48.1
>8971	9	2.3

Others

*: -unemployed, solider, driver, retire and artist.

### Clinical, behavioral, and obstetric characteristics

Of the total participants, 30 (7.4%) had a family history of hemorrhoids and one fourth 102 (25.3%) had a history of alcohol intake. Among female study participants, the majority of them 153 (74%) gave at least one birth. Out of the total study participants, 96 (24%) had constipation and 151 (37.4) had a history of taking a high fiber diet (Table 2).

**Table 2:** Clinical, behavioral and obstetric characteristics of adult patients visiting surgical OPD at the UOG Comprehensive Hospital, Ethiopia, 2020 (n = 403).

Variable	Frequency	Percentage (%)
**Family history of hemorrhoids**		
Yes	30	7.4
No	373	92.6
**Smoking**		
No smoking	395	98.0
Previously smoking	6	1.5
Currently smoking	2	0.5
**Alcohol intake**		
No alcohol	301	74.7
Previous alcohol intake	32	8.0
Current alcohol intake	70	17.3
**Parity**		
Nulliparous	54	26.0
Prim parous	22	10.7
Multi parous	68	32.9
Grand multipara	63	30.4
**Constipation**		
Yes	96	23.9
No	307	76.1
**Physical exercise**		
Yes	46	10.4
No	361	89.6
**High fiber diet intake**		
Yes	151	37.47
No	252	62.53
**Fat meal**		
Yes	56	13.9
No	347	86.1
**History of repeated diarrhea**		
Yes	18	4.47
No	385	95.53
**Blood pressure**		
<130/80mmhg	269	66.75
>130/80	134	33.25
**BMI**		
14–17.9	58	14.39
18–24.9	311	77.17
25–29.9	27	6.70
30–34.9	7	1.74

### Prevalence of hemorrhoids

**Fig 1:**
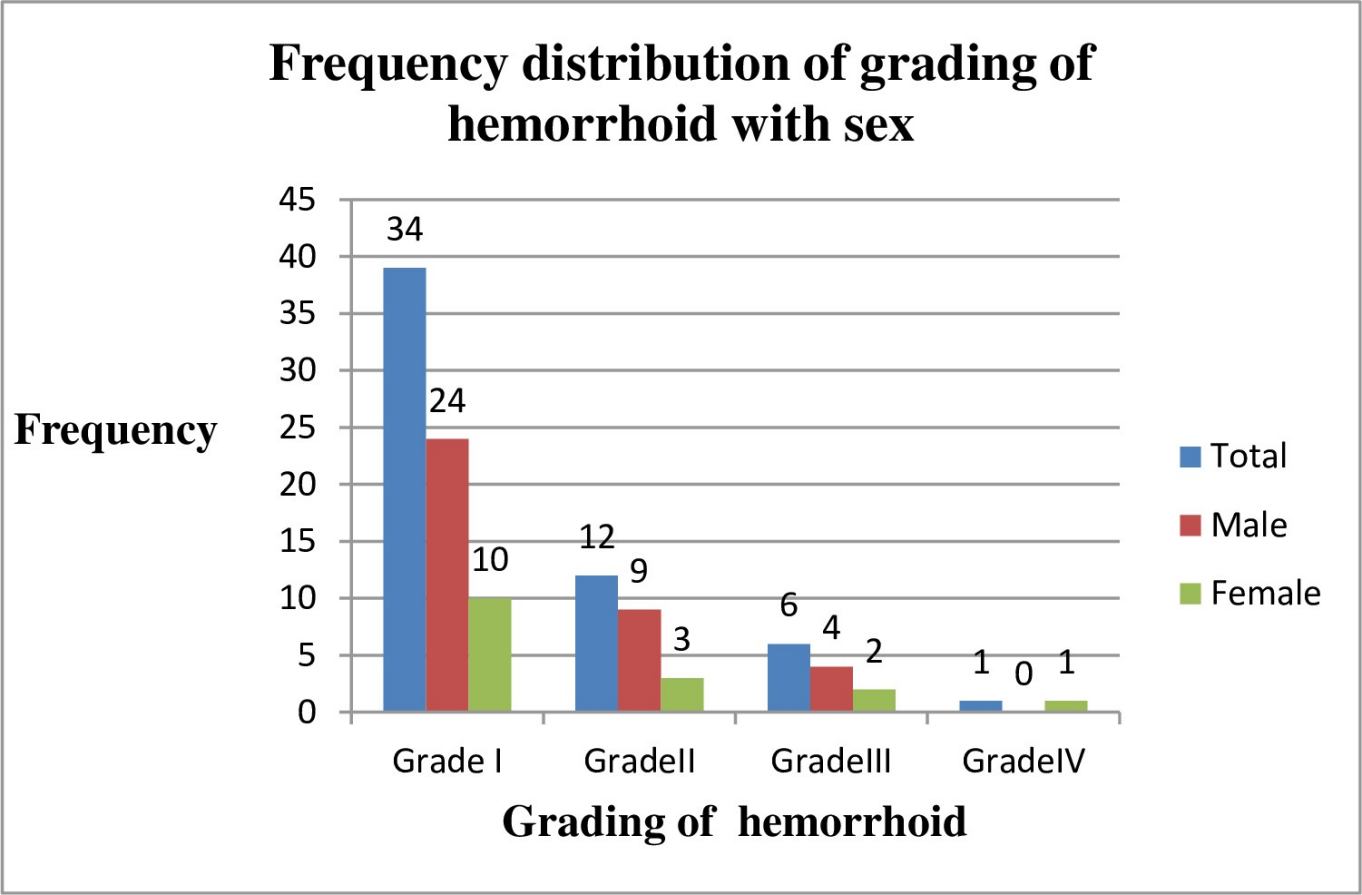
Frequency distribution of grading of hemorrhoid with sex of adult patients visiting surgical OPD at the UOG Comprehensive Hospital, Ethiopia, 2020.

The result of this study revealed that among 403 study participants 53 had hemorrhoids with an overall prevalence of 13.1% (95%CI; 10.1, 16.8). Participants having hemorrhoid were classified as grade I to IV and the prevalence of grade I, II, III, and IV were 34 (64.1%), 12 (22.7%), 6 (11.3%), and 1 (1.9%), respectively. The prevalence of hemorrhoids among male and female participants was 18.8% (95% CI: 13.6, 25.0) and 7.7% (95%CI: 4.48, 12.4), respectively. Out of the total of hemorrhoid cases that occurred among females, 11 (68.7%) of them were diagnosed from those who gave one and above birth (Fig 1).

### Factors associated with hemorrhoids

From the multivariable logistic regression analysis constipation and BMI had a significant association with the occurrence of hemorrhoid. The odds of having hemorrhoid was 4.32 times higher among participants who had constipation as compared to their counterparts (AOR = 4.32, 95%CI; 2.20, 8.48). The patients who had BMI ≥25 kg/m^2^ were 2.6 times higher odds of having hemorrhoid as compared to BMI < 25 kg/m^2^ (AOR = 2.6, 95%CI; 1.08, 6.23) (Table 3).

**Table 3:** Multiple logistic regression output for the factors associated with hemorrhoids among adult patients visiting surgical OPD at the UOG Comprehensive Hospital, Ethiopia, 2020 (n = 403).

Variable	Hemorrhoids		Crude OR(95%CI)	Adjusted OR(95%CI)	P-value
	Yes	No			
**Age**					
19–45	28	239	1	1	
46–84	25	111	1.92(1.07, 3.44)	1.02(0.52, 2.03)	0.93
**Sex**					
Male	37	159	0.35(019,0.67)	1.82(0.91, 3.66)	0.08
Female	16	191		1	
**Average monthly income**					
<1210	19	181	1	1	
1211–8970	32	162	1.88(1.02,3.44)	1.52(0.76, 3.03)	0.22
>8971	2	7	2.72(0.52,14.0)	4.56(0.76, 27.1)	0.09
**Constipation**					
Yes	26	70	5.05(2.68, 9.51)	4.32(2.20, 8.48)	0.000
No	21	286	1	1	
**Fiber diet intake**					
Yes	29	55	8.81(4.58, 16.9)	0.51(0.22 1.15)	0.105
No	18	301	1	1	
**BMI**					
**<24.9 kg/m**^**2**^	41	320	1	1	
**>259 kg/m**^**2**^	12	30	3.12(1.48,6.57)	2.60(1.08, 6.23)	0.032
**Blood pressure**					
**<130/80mmhg**	29	240	1	1	
**> = 130/80mmhg**	24	110	1.80(1.00, 3.24)	1.48(0.77, 2.84)	0.234

AOR: Adjusted Odds Ratio; COR: Crude Odds Ratio; CI: Confidence-interval, BMI: Body mass index, mmhg: mili meters of mercury, kg/m^2^: kilogram per meter square.

## Discussion

The present study was conducted to determine the prevalence of hemorrhoids in adult patients visiting surgical OPD at the UOG Comprehensive Hospital, Ethiopia, and to define associated risk factors. Constipation and being overweight found to be significantly associated with hemorrhoids.

In this study, the prevalence of hemorrhoids was found to be 13.1%. The result is consistent with a study conducted in Israel 16% (13) and Korea 14.4% (14). However, it is lower than the study from Australia and Egypt which reported the prevalence to be 38.9% and 18% respectively [12, 15]. In Australia, participants were from colorectal cancer screening and the investigation was conducted in multi-centered area. In a way, hemorrhoids and colorectal cancer have similar symptom which may increase the prevalence in Austria study. Similarly, study conducted in Egypt included those patients who came for colonoscopic examinations and patients with anorectal disease which may contribute to the rise of the prevalence of hemorrhoid. However, in our study we considered merely patients who visited surgical OPD.

In our study, study subjects with constipation were more likely to have hemorrhoids as compared to their counterparts. Similarly, studies conducted elsewhere supported the notion of significant contribution of constipation to the induction of hemorrhoid [1, 22–24]. This could be due to degeneration of the supportive tissue in the anal canal and tear of elastic supportive tissue due to prolonged straining during defecation and hard stool Subsequently, causing a distal displacement of anal cushions and development of hemorrhoid [25, 26]. Passage of hard stool and increased intra-abdominal pressure could also obstruct venous return, resulting in engorgement of the hemorrhoidal plexus and arteriovenous anastomoses of the anorectal junction this leads to the development of hemorrhoid [27].

The current study found that being overweight increased the odds of having hemorrhoids. The notion of our study is supported by other studies done elsewhere [12, 14, 28]. This could be attributed to an increase in the intra-abdominal pressure due to the high body weight and visceral fats which are thought to give rise to the venous congestion of the distal rectum [14]. Obesity will induce the release of inflammatory cytokines and acute phase proteins which will eventually activate the innate immune system and affect metabolic homeostasis, which contributes to the formation of hemorrhoids [14].

This study helps us to know the burden and possible risk factor of the disease and may allow us to easily identify individuals at risk of hemorrhoids and to provide early diagnosis, prevention measures, and appropriate interventions. However, there are some limitations of this study such as it could not establish a cause-effect relationship because of the cross sectional nature of the study design. In addition, this study was an institution based and the findings may not fully reflect the entire population and also possible that recall bias may have been introduced. The study did not assess the frequency of fiber diet intake based on the recommendations of WHO.

## Conclusion

Hemorrhoid is found to be the common health problem among surgical patients and its prevalence was higher in male subjects. Constipation and being overweight were found to increase the odds of having hemorrhoids. Screening for early identification and intervention of hemorrhoids, especially for risk groups is better to be practiced by health professionals. We recommend every individual to maintain their normal body weight and avoid any risk that can cause constipation. Further, community based study should be conducted on the burden of hemorrhoid in Ethiopia.

## Supporting information

S1 File (PDF)

## Abbreviations

BMI: Body mass index
IAP: Intra-abdominal pressure
BP: blood pressure
OPD: Outpatient department
UOGCSH: University of Gondar Comprehensive Specialized Hospital
USA: United State of America
