# Supplementary material for: Prevalence and associated factors of hemorrhoids among adult patients visiting the surgical outpatient department in the University of Gondar Comprehensive Specialized Hospital, Northwest Ethiopia
Source: PLoS One. 2021 Apr 20;16(4):e0249736. doi: 10.1371/journal.pone.0249736 (PMC8057569; doi:10.1371/journal.pone.0249736)
Supplement: S1 File — (PDF) [file pone.0249736.s001.pdf]

Consent form

Questionnaire for a research on prevalence and associated factors of hemorrhoids among adult surgical patients at university of Gondar compressive specialized hospital, North West Ethiopia in 2020.

Confidentiality and consent

Hello, my name is **_______**; I am one of the data collectors in this study. The study is intended to assess prevalence and associated factors of hemorrhoids. To attain this purpose, your honest and genuine participation by responding to the questions prepared is very important and highly appreciated. I would like you to ask some personal questions. Your answers are completely confidential. No one will be told what you said in connection to your name. You don't have to answer any question if you do not want to and you can stop the interview at any time. However, your honest answer to these questions will help us to better understand the situation and will contribute to our study. We would greatly appreciate your help in participating in this study, would you be willing to participate.

If yes, proceed.

If no, thank you and stop here.

____

(Signature of the interviewer certifying that respondent has given informed consent verbally

Annexes 1: Questionnaire (English version)

Questionnaire for a research on prevalence and associated factors of hemorrhoids among adult surgical patients at university of Gondar compressive specialized hospital, North West Ethiopia in 2020.

Interviewer's Name___________________________________ Date of interview_________

Supervisor's Name_____________________________________ Questionnaire No________

A. Socio-demographic characteristics			
**No**	**Question**	**Response**	**Remark**
101	Sex	1) Male<br>2) Female	
102	Age	_______ years	
103	Place of residence	1) Urban<br>2) Rural	
104	Occupation of the study participant?	1) Civil servant<br>2) Merchant<br>3) Farmer<br>4) Housewife<br>5) Self employed<br>6) Daily laborer<br>7) Student<br>8) Others	Others specify ______
105	Religion of the study participant?	1) Orthodox<br>2) Muslim<br>3) Protestant<br>4) Catholic<br>5) Others	Others specify ______

106	Education status of the study participant?	1) Unable to read &write<br>2) Able to read & write<br>3) Primary education (grade 1–8)<br>4) Secondary education (grade 9–10)<br>5) Preparatory (grade 11–12)<br>6) College or above	
107	Marital Status of the study participant?	1) Single<br>2) Married<br>3) Divorced<br>4) Widowed<br>5) separated	
108	Average monthly income	______________	
B. Clinical, obstetric and behavioral factors			
201	Do you have family history of hemorrhoid?	1. Yes<br>2. No	
202	Do you eat fruits and vegetables?	1. Yes<br>2. No	
203	If yes, how many times do you eat fruits and vegetables in a week?	______________________ days	
204	Do you eat fat meals?	1. Yes<br>2. No	
205	If yes, how many times do you eat fat meals in a week?	_____________days	
206	Do you have infrequent stool, difficulty in defecation or both at least for previous 3 months?	1. Yes<br>2. No	

207	Do you have repeated diarrhea?	1. Yes<br>2. No	
208	How many children do you have? for women only	___________________	
209	Do you smoke cigarettes?	1) Never<br>2) I used to smoke<br>3) I currently smoke	
210	How would you describe your alcoholic habit?	1) Never drank alcohol<br>2) Previous alcoholic<br>3) Current alcoholic	
211	Do you work physical exercise?	1. Yes<br>2. Sometimes<br>3. No	
212	Have you diagnosed of hypertension by physician Previously?	1. Yes<br>2. No	
213	Do you use the anti-hypertensive drugs?	1. Yes<br>2. No	
	**C. Physical examination**		
301	Blood pressure measurement first reading	___________mmHg	
302	Second reading	____________mmHg	
303	Third reading	____________mmHg	
304	Wight	___________kg	
305	Height	____________cm	
306	Body mass index	___________kg/m^2	
307	Hemorrhoids	1. Yes<br>2. No	

Annex- 2: Amharic version questionnaire

ቃለ-መጠይቅ

በጎንደር ዩኒቨርሲቲ ጤና ሳይንስ ኮሌጅ ከሚመጡ የቀዶ ህክምና ታካሚወች ላይ በኪንታሮት በሽታ ስርጭት ና ተያያዥ ምክንያቶች በተመለከተ ለማጥናት የተዘጋጀ ቃለ-መጠይቅ፡

ቃል መጠይቅ የሚያደርገው ስም -----------------

ቃል መጠይቅ የተደረገበት ቀን ------------------

የጥናቱ ተሳታፊ የሚስጥር ቁጥር ------------------

ሀ. ማህበራዊ መስተጋብር ጥያቄወች			
ተ.ቁ	ጥያቄ	መልስ	ምርመራ
101	ጾታ	1. ወንድ<br>2. ሴት	
102	እድሜ /በአመት/	________	
103	የመኖርያ ቦታ	1.ከተማ<br>2.ገጠር	
104	ስራ	1. አርሶ አደር<br>2. ነጋደ<br>3. የመንግስት ተቀጣሪ<br>4. የቤት እመቤት<br>5.ተማሪ<br>6.የቀን ሰራተኛ<br>7.ስራ አጥ<br>8.ሌላ(ይጥቀሱ)	
105	ሃይማኖት	1. ኦርቶዶክስ<br>2. ሙስሊም<br>3. ፕሮቴስታንት<br>4. ካቶሊክ<br>5. ሌላ (ይጥቀሱ)	
106	የትምህርት ደረጃ	1.ማንበብናመጻፍ የማይችል<br>2.ማንበብናመጻፍ የሚችል<br>3. የመጀመሪያ ደረጃ /1-8/<br>4. ሁለተኛ ደረጃ /9-12/ክፍል<br>5. ኮሌጅና ከዚያ በላይ	
107	የጋብቻ ሁኔታ	1.አግብታ/ አብራ የምትኖር/የሚኖር<br>2.አግብታ/ቶ የፈታች/ታ<br>3.ባሏ/ሚስት የሞተባት/የሞተበት<br>4.ያላገባ/ች<br>5.ሌላ ካለ ይጥቀሱ-------------	
108	አማካኝ የወር ገቢ	________________________________	

ሰ. ከ ኪንታሮት በሽታ ጋር የተያያዙ ጥያቄወች			
201	ከቤተሰብወት ውስጥ ኪንታሮት በሽታ ያለው አለ ?	1. አለ<br>2. የለም	
202	ቅጠላቅጠል ና ፍራፍሬ ይመጋባሉ?	1. አወ<br>2. አልመገብም	
203	አወ ከሆነ መልስወት ፤በሳምንት ምን ያህል ቀን ቅጠላቅጠል ና ፍራፍሬ ይመጋባሉ?	_______ቀን	
204	ቅባት የበዛበት ምግብ ይመጋባሉ?	1. አወ<br>2. አልመገብም	
205	አወ ከሆነ መልስወት ፤በሳምንት ምን ያህል ቀን ቅባት የበዛበት ምግብ ይመጋባሉ?	________ቀን	
206	ሽንት ቤት በሚወጡበት ወቅት ሰገራ ለመውጣት ያስቸግርወታል ወይም የሆድ ድርቀት በዚህ 3 ወር ውስጥ አለወት ?	1. አወ<br>2. የለም	
207	በተደጋጋሚ ተቅማጥ ያምወታል?	1. አወ<br>2. የለም	
208	ስንት ልጆች አለወት? ለሴቶች ብቻ	---------------------------	
209	ሲጋራ ያጨሳሉ?	1. አጭሼ አላውቅም<br>2. አጨስ ነበር<br>3. አጨሳለው	
210	አልኮል መጠጥ ይጠጣሉ?	1. አልኮል ጠጥቸ አላውቅም<br>2. አልኮል እጠጣ ነበር<br>3. አልኮል እጠጣለው	

211	የአካል ብቃት እንቅስካሴ ያደርጋሉ?	1. አወ<br>2. አልፎ አልፎ አደርጋለው<br>3. አላደርግም	
212	የደም ግፊት በሽታ አለብወት ተብሎ በሃኪም ተነግሮውት ያውቃል?	1. አወ<br>2. የለም	
213	በሃኪም የታዘዘ የደም ግፊት መድሃኒት ይወሰዳሉ?	1. አወ<br>2. የለም	
ሐ. አካላዊ ምርመራ			
301	የ ደም ግፊት የመጀመሪያ ንባብ	___________mmHg	
302	ሁለተኛ ንባብ	____________mmHg	
303	ሶስተኛው ንባብ	____________mmHg	
304	የስውነት ክብደት	___________kg	
305	ቁመት	____________cm	
306	የሰውነት መጠን መለክያ	___________kg/m^2	
307	ኪንታሮት በሽታ	1. አለ<br>2. የለም	
